# Pneumonia care and the nursing home: a qualitative descriptive study of resident and family member perspectives

**DOI:** 10.1186/1471-2318-6-2

**Published:** 2006-01-23

**Authors:** Soo Chan Carusone, Mark Loeb, Lynne Lohfeld

**Affiliations:** 1 Department of Clinical Epidemiology and Biostatistics, McMaster University, Hamilton, Canada; 2 Department of Pathology and Molecular Medicine, McMaster University, Canada; 3 Program for Educational Research and Development, McMaster University, Canada

## Abstract

**Background:**

Nursing home residents are frequently sent to hospital for diagnostic tests or to receive acute health care services. These transfers are both costly and for some, associated with increased risks. Although improved technology allows long-term care facilities to deliver more complex health care on site, if this is to become a trend then residents and family members must see the value of such care. This qualitative study examined resident and family member perspectives on *in situ *care for pneumonia.

**Methods:**

A qualitative descriptive study design was used. Participants were residents and family members of residents treated for pneumonia drawn from a larger randomized controlled trial of a clinical pathway to manage nursing home-acquired pneumonia on-site. A total of 14 in-depth interviews were conducted. Interview data were analyzed using the editing style, described by Miller and Crabtree, to identify key themes.

**Results:**

Both residents and family members preferred that pneumonia be treated in the nursing home, where possible. They both felt that caring and attention are key aspects of care which are more easily accessible in the nursing home setting. However, residents felt that staff or doctors should make the decision whether to hospitalize them, whereas family members wanted to be consulted or involved in the decision-making process.

**Conclusion:**

These findings suggest that interventions to reduce hospitalization of nursing home residents with pneumonia are consistent with resident and family member preferences.

## Background

The demand for long-term care in facilities is increasing in response to changing demographics and social values. As of 2000, an estimated 46 percent of Americans 65 years old will spend time in a nursing home before they die. By 2020, the total number of older adults using nursing home care in the United States is expected to more than double [1].

The functional dependence and clinical complexity of health problems that long-term care facility (LTCF) residents have are also increasing. In 1997, the United States' National Nursing Home Survey found that 75% of elderly nursing home residents needed help with three or more activities of daily living (bathing, dressing, eating, transfer from bed to chair, toileting), and that 44% had difficulty with both bowel and bladder continence [2]. Although many LTCF residents are currently transferred to hospital for diagnostic tests or to receive acute medical services, fiscal pressures, improved technology, and complications associated with hospitalization suggest that more medical care should be provided in nursing homes.

Pneumonia and other lower respiratory tract infections (LRIs) are a major cause of morbidity and mortality among nursing home residents. They are also the leading reason for their hospitalization. One Canadian study found that nearly one-third of all LTCF residents with pneumonia were hospitalized [3]. Recent research suggests that residents with pneumonia at a low- to medium-level mortality risk may be managed safely in a LTCF for less cost [4,5].

Some researchers have argued that the provision of health care cannot be decontextualized from the environment in which it is provided. As such, the locus of care is an important issue. There are also a variety of perspectives to understand in relation to this issue – specifically that of older adults, their families, friends, and health care providers [6]. Although the decision about where and when LTCF residents should receive care is no longer solely in their control, it is important to understand their preferences for care.

Few studies have examined the care preferences of LTCF residents and their families and most of this work has been done with the use of surveys to assess the views of well people in response to hypothetical situations. Two such studies have found that nursing home residents generally prefer hospital-based care [7,8]. Kleinman [9], however, suggests that generic models of health-related behaviors are very different from responses to specific illness episodes experienced by a person, and that the latter are essential to understanding help-seeking behaviors for sickness. The objective of this study was to learn if LTCF care for pneumonia is consistent with resident and family preferences using a qualitative descriptive study design.

## Methods

This study was part of a multi-centred randomized controlled clinical trial that tested the effectiveness and utility of using a protocol for treating nursing home-acquired pneumonia. The protocol listed signs and symptoms of pneumonia and directed staff to follow a treatment pathway that included criteria for deciding the appropriate locus of care (LTCF vs. hospital). Twenty nursing homes in southern Ontario were matched by size and one member in each pair was randomly allocated to use the clinical pathway. The other facility continued to follow normal care practices to diagnose and treat pneumonia. From November 2003 to June 2004, research nurses approached primary decision makers (residents or family members of residents who were deemed incapable of making informed decisions regarding their care) to participate in the qualitative study.

### Sampling and recruitment

Inclusion and exclusion criteria for the clinical trial are summarized in Table 1. After 30 days of follow up in the clinical trial study, residents with pneumonia and family members were invited by a clinical trial study nurse to participate in the qualitative study. Our aim was to enrol information-rich participants, or people who can best describe the experience under study (purposeful sampling) [10]. As a result, study nurses were asked to only invite residents they deemed capable of remembering and discussing care provided for a recent case of pneumonia (residents), or family members who were most directly involved in decision-making for residents unable to speak about their own care. Residents and family members who indicated they were willing to participate in this study gave consent to have their names released to the researcher (SCC) who then explained the study to them prior to obtaining informed consent. Although our goal was to recruit individuals until saturation of the main themes was achieved, we were limited by the number of eligible participants enrolled in the clinical trial during the data collection period. However, a strong consensus among participants' views on the major topics raised during data collection was achieved.

**Table 1:** Inclusion and exclusion criteria of the clinical trial*

**Inclusion Criteria**	**Exclusion Criteria**
Have 2 or more of the following signs or symptoms: • New or increased cough • New or increased sputum production • Fever (>38°C) • Pleuritic chest pain • New or increased findings on chest examination	1. Residents not expected to live longer than 30 d (from enrolment) 2. Residents who have had a previous anaphylactic or allergic reaction to quinolones 3. Residents who have not provided consent 4. Residents with advanced directives stating that they are not to be transferred to hospital for treatment

*This qualitative descriptive study was nested within a much larger multi-centred clinical trial.

### Data collection

Data were collected by the researcher (SCC) in one-time, individual, semi-structured interviews with residents (n = 6) and family members (n = 8). All of the resident interviews were performed in one of four nursing homes. Family member interviews were performed in nursing homes, at coffee shops, or by telephone. Interviews lasted between 20 and 90 minutes, depending on the participants' ability to express themselves. All but one interview was tape recorded and transcribed verbatim for accuracy. Data were collected in the one non-taped interview by extensive note taking during and immediately after the interview. Interviews focused on four themes: participants' experience with a recent case of pneumonia, preferred locus of care for pneumonia (hospital or nursing home), perceived differences between LTCF- and hospital-based care, and what constitutes 'good care'. Preliminary analysis of the first four transcripts revealed an important but unanticipated theme: participants' desired involvement in treatment decision-making. This topic was therefore included in subsequent interviews. The interview guides for the resident and family member interviews were similar. The only differences were that resident interviews probed for more information about the actual care that residents received, and family member interviews addressed both family members' actual preferences as well as their views on the preferences and experiences of the residents they spoke about (See Table 2 for the final version of the resident interview guide).

**Table 2:** Interview guide for residents

**Diagnosis** Thinking back to when you were sick, what sort of symptoms did you have? Who first told you that you had pneumonia? How did you feel when they told you that you had pneumonia? Have you had pneumonia before?
**Treatment** What sort of treatment did you receive? How often did you see the doctor? What could have made the care that you received better?

**Quality of Care** To you, what is the most important aspect of care? What makes you feel like you are being well taken care of?

**Preferences for care** If you had a choice, where would you have preferred to receive care (in the nursing home or in the hospital)? Would you like to be asked where you would like to receive treatment? Or, would you prefer the doctor or nurses to make the decision on their own?

**Differences between hospital and nursing home** What sort of differences do you see between the care that you would receive here versus the care that you would receive in the hospital? What would make you think that you have to go to hospital?

### Rigour and credibility

Numerous steps were taken to ensure that the findings were faithful to the participants' descriptions and interpretations (credible), and that the research process could be followed by another researcher (rigorous). All interviews were conducted by the same person (SCC) to ensure continuity across interviews (reduce bias). Following the recommendations of Miller and Crabtree [11], the researcher made reflective journal entries throughout the study. Two types of triangulation were used in this study. Data were collected from both LTCF residents and from residents' family members (multiple sources of data), and two researchers independently coded transcripts and compared their findings (multiple researchers). A third researcher, with extensive clinical and research experience, was consulted at all stages of the study (peer review).

### Ethical considerations

Informed consent was received from all participants prior to conducting an interview. Individuals were assured that their care would not be affected in any way by their decision about participating in the study. None of the study nurses or researchers worked for a nursing home enrolled in the study, and did not provide care outside of the study. This study was approved by the research ethics board at St Joseph's Hospital in Hamilton, Ontario, Canada.

### Data analysis

Following standard practice, audiotapes produced during each interview were transformed into verbatim written accounts (transcripts) by a professional typist. The researcher (SCC) then compared the written and audiotaped versions of each interview in order to correct transcription errors. Data from earlier transcripts were analyzed concurrently with ongoing data collection [10,12] in order to ensure that emerging themes could be further pursued in later interviews. Analysis followed a five-phase process [12]. In phase one (description), transcripts were read in their entirety without coding the data and reflexive journaling was used to gain an overview or overall sense of the views of study participants. Phases two and three (organizing and connecting data) involved more detailed transcript review to identify key phrases and words, and then pattern coding [13] or clustering them into themes, followed by data reduction and linking across clusters. In phase four (corroborating/legitimating), two researchers (SCC & LL) individually coded the transcripts and compared their findings to reach consensus about disconfirming evidence and alternative explanations. Phase five (representing the account) involved highlighting results with supporting quotes (linking findings to the data), and interpreting the findings in light of relevant literature.

### Presenting results

Following standard procedures for reporting interview data [14], exemplars, or typical statements made by participants, are presented initalics to support conclusions drawn by the researchers. The views of LTCF residents and family members are presented separately to aid comparisons across these two groups in the discussion section of this paper. Participants are identified by a letter ("R" = resident, "FM" = family member") and a number based on the sequence in which interviews were conducted. For example, "R4" is the fourth resident we interviewed. Minimal editing was done to preserve authenticity while ensuring readability [15]. Ellipses (...) were used where irrelevant information was deleted from a quote. Where necessary, clarifying information was added to a participant's words in square brackets ([ ]).

## Results

### Participants

Participants included six residents and eight family members. All of the residents were females between the ages of 76 and 93 years (mean age = 84 years). Residents varied greatly in their functional status, as measured by a modified-Barthel Index used to rate status on 10 daily functions for a summary score that ranges from 0 (full dependence) to 20 (full independence) points. Four of the residents were extremely dependent (Barthel Index < 10, range: 0 to 9) and two were moderately independent (Barthel Index 10–20). Two residents had been hospitalized for pneumonia while enrolled in the clinical trial. Half of the family members were female (2 wives and 2 daughters or daughters-in-law) and the other four were sons of LTCF residents. The seven residents they spoke about (5 of them female) ranged in age from 84 to 96 years (mean age = 91 years) and scored very low on the Barthel Index (0–12). One of the residents they spoke about had been hospitalized for pneumonia and died upon return to the LTCF. All the participants were recruited from five nursing homes (2 for-profit, 3 not-for-profit) with 100–250 beds (see Table 3 for a summary of participant characteristics).

**Table 3:** Participant characteristics

**ID (relationship)**	Sex*	Age*	Hospitalized*	Barthel*^a^
R1	Female	76	Yes	6
R2	Female	84	No	9
R3	Female	93	No	14
R4	Female	86	No	17
R5	Female	84	Yes	0
R6	Female	83	No	7
FM1 (son)	Female	96	Yes	3
FM2 (daughter)	Female	88	No	8
FM3 (son)	Female	98	No	3
FM4 (daughter-in-law)	Female	93	No	12
FM5 (son)	Male	88	No	0
FM6 (wife)	Male	88	No	0
FM7 (son)	Female	84	No	0
FM8 (wife)	Male	92	No	10

* Characteristics of resident

^a ^Modified Barthel Index: 0 indicates complete dependence and 20, complete independence.

Participants readily spoke about the four topics raised in the interviews. Both residents and family members preferred that care be provided in the nursing home (when possible), although for slightly different reasons. They also had different views on how decisions about locus of care should be made.

### Preferred locus of care

Both residents and family members largely preferred that pneumonia be treated in the nursing home. This appears to be a function of both their beliefs about pneumonia and how they define good care. Both groups of participants believed that hospital care is clearly necessary for some conditions (e.g. fainting, broken bones, operations, and heart problems) but not for pneumonia (*'I don't want to go to hospital *[for pneumonia]. *If you need an operation, that's different' *[R3, page 5]).

*Residents: *Although all residents in the study had been diagnosed with pneumonia or a LRI, and two of them had even been hospitalized for this condition, they were generally not very concerned by such a diagnosis. Some residents referred to their illness simply as a 'cold' or the 'flu' and seemed to have had trouble believing it was pneumonia (*'I thought, "Oh, no, I haven't got pneumonia!" I was just surprised that I had it, or that I was supposed to have it.' *[R5: page 1]). They generally felt that pneumonia could be cared for in the nursing home (*'I don't want to be in the hospital again... I know they have taken people from here to the hospital. I guess when they get pretty bad... *[but] *I don't think they could get any better treatment then we get here.' *[R2, page 7]).

*Family members: *Family members were more concerned than residents about the diagnosis of pneumonia, recognizing that it could be a serious illness in the elderly. However, many of them still felt that it can usually be managed on-site:

*'In the elderly*, [pneumonia] *is quite a serious thing. If it's caught early, which it is usually in the nursing home, I think it's better to be treated here. With the antibiotics that they have now, and the *[fact that the] *nurse came in every day and checked her every day *[for] *the oxygen level, the care was terrific.' *[FM4, page 2]

Despite preferring on-site care for pneumonia, some family members acknowledged that they were not sure what level of acute care a LTCF could provide:

*'I think *[my mother] *would get better care *[in the nursing home] *than she probably would in the hospital, except she would not be very ill. Because when she is very ill, I don't think they would be able to look after her *[here]. *They haven't got the facilities. I don't think they're geared to do that.' *[FM2, page 5]

In some cases, family members acknowledged that even if their loved one was very sick, it was not necessarily desirable to provide care in hospital:

*'Let's be honest about it. He's 88. He's been on the verge of death several times. The man has no quality of life at all; zero... Why are we going to use hospital resources when all we really want to do is make him comfortable in his last hours or months or years, or whatever he's got left? I don't think hospital intervention is going to improve his quality of life at all.' *[FM5, page 2]

### Defining good quality care

Both residents and family members expressed the view that residents with pneumonia can receive more comfort and personal attention in a LTCF than in hospital, and therefore preferred *in situ *care.

*Residents: *For these participants, signs of good care included paying attention to a resident's comfort, as well as personal attention, interest, and time given by nurses (*'We've got some real nice nurses here. They really care.' *[R2, page 6]; *'They always take time to listen to you if you tell them there is something wrong' *[R3, page 6]). The only treatment-related aspects of care that residents specifically mentioned involved easing discomfort (*' *[In this facility] *they usually give me Tylenol and good care. *[They] *see that I'm looked after alright and comfortable' *[R2, page 5]).

Although residents were more hesitant than family members about expressing any negative opinions about care, several of them were able to clearly identify problems, such as extremely busy staff or their lack of availability (*' *[The nursing home] *is better, I think. I think they have more time here – not that they have a lot of time, but they seem to have more time than in the hospital.' *[R5, page 4]), including the lack of availability of some doctors (*'We call Dr. *[So-and-So] *"the Phantom": He goes to the office and then shoots down the hall!' *[R1, page 1]). Unlike family members, residents generally brought up such issues with an understanding or accepting attitude (*'You just put up with what you have to' *[R5, page 4]).

*Family members: *These participants, like the residents we spoke with, thought that a caring attitude and personal attention from nurses are signs of good care:

*'Well*, [in] *the hospital, if I remember correctly, they don't have the time. The poor nurses, they just don't have the time. Everything is sort of rush, rush, rush, and they don't really listen. *[My mother] *went in when she hurt her knee... and we were there from 11 at night until the next morning. At eight o'clock at night she came home... *[In the hospital], *they're so rushed that *[the nursing home] *is better. *[It] *is more personal care.' *[FM4, page 1]

Nurses' attitudes and personality were also important aspects of care (*'My mother liked a friendly person, somebody she could kid around with and joke *[with]' [FM1, page 7]). Family members also wanted to be reassured about their loved ones' care (*'Where she is now, I have no qualms leaving my mom there because I think it's one of the best homes there are... Before she was in *[another nursing home and] *I wouldn't have left my mom there' *[FM2, page 2]; *'She gets very good care. It's a very good nursing home. It's one we chose' *[FM7, page 2]).

Although family members generally preferred that care be given in the nursing home, they were much more critical than the residents of the care provided in LTCFs. They also more readily identified benefits of hospital-based care (*'Definitely there is no doctor here *[at the nursing home] *on a permanent basis, so the hospital would provide better care, once admitted' *[FM3, page 2]; ' [In the hospital] *it's not a question of taking blood, sending it to the laboratory and having somebody come back three days later... they immediately check it and they know exactly *[what is going on]' [FM1, page 3]).

The number one complaint made by family members about nursing home care was that the staff are too busy and, in some cases, personal care is inadequate (*'Sometimes he *[urinates] *in bed. It's not a pleasure for him... It takes so long before *[the nurses] *come... They're always in a hurry. I can see it's because they are too short of staff' *[FM6, page 3]). Although not a major theme, some concern was also expressed over the level of training that staff receive:

*'In a nursing home, 90-something percent of *[the residents'] *contacts are with the lowest paid, medically unqualified people... Some of the health care aides are lovely, you know, they're really nice. But ... the training is very short, as far as I can determine.' *[FM1, page 7]

### Reasons for preferring nursing home care for pneumonia

Both residents and family members identified other factors associated with their preference for LTCF-based care. The most central of these was the view that the LTCF is a resident's 'home'.

*Residents: *Many of these participants stated that they did not want to go to hospital even though one can receive good care there. The reasons they gave for this view were that hospitals were busier, more isolating, and more confining than nursing homes (*'You are hooked up to everything... oxygen, the heart machine... You don't know the nurse that's hard.' *[R1, page 2]; *'I think it's more comfortable here *[in the nursing home] *than in the hospital. There's not as much going on in the halls as in the hospital.' *[R5, page 4]). The LTCF had become their home (*'Everybody knows me. I am friends with everybody.' *[R6, page 3]; *'I think most people like it back in the nursing home. Hospital is hospital. I'm not so good in the hospital. You want to be in your own room.' *[R4, page 7]). Some residents also mentioned the inconvenience that going to hospital caused family members.

*Family members: *Family members also preferred that their loved ones receive pneumonia care in the nursing home, although they were more willing to accept that hospitalization might be necessary. Many of them indicated that the LTCF is the senior's home and the benefits of being in familiar surroundings (*'I think the same surroundings really helps the elderly patient; *[my mother] *would be still in her own room and her own bed. Even in my mother's case, where she now has this dementia, she talks about her room like her home' *[FM3, page 3]). Some family members also expressed the view that *in situ *care was preferable because of the difficulties that residents have adjusting to life in the hospital (*'To transfer *[my mother] *to hospital and get use to the hospital environment, I think is more detrimental... *[even though] *I think they would get more superior treatment in the hospital... and the medical staff assessment there would be far superior than in the nursing home' *[FM7, page 1&3]). This was particularly the case if the LTCF resident had dementia:

*'I think the confusion is more in the hospital*. [The last time my mother was in the hospital] *the nurse said, "Can you stay to just keep your eye on her?"... *[My mother] *was going to go home and therewas just no two ways about it. They couldn't keep her there. She had the whole floor in an uproar.*' [FM2, page 2]

From a personal perspective, several family members also explained that it is more convenient for them if their loved ones receive care in the LTCF:

*'The family's more comfortable *[in the nursing home], *I think... One, there's parking; two, I don't have to deal with, "Can I go in *[or] *can I not?"... It's more familiar. It's the central place. My mother is already there. We don't have to lug her back and forth *[to see my father].' [FM5, page 3]

### Making the decision about locus of care

Although both residents and family members preferred that pneumonia care be provided *in situ*, they differed in their opinion of how they would like the decision to hospitalize to be made.

*Residents: *These participants often admitted that when they were ill, they were *'too sick to care' *and *'didn't care what they did' *[R3, page 5]. Even when not so overwhelmed, residents generally wanted their doctors to make the treatment decisions for them. Several residents equated voicing any preferences about care to their doctors with complaining or with being difficult or bossy (*'Just tell *[me what to do], *not *[that] *I would say, "No, I don't like to go *[to hospital] *" and be bossy. That is not my person *[ality], *not at all.' *[R4, page 4]). In one instance, a resident admitted that she had voiced her preference to the nurse, but explained she would never talk that way to her doctor.

*Family members: *In contrast, when possible, family members wanted nursing home staff to suggest treatment options and discuss their loved one's care with them before transferring the LTCF resident to hospital (*'I expect them to be the professional on the job and make suggestions. If I have a problem with their suggestion, they can always go to option two with me.' *[FM5, page 2]).

Unlike the residents, family members were quite willing to voice their care preferences. Several of them stated that they were the ones who ultimately made treatment decisions (*'I would rely on their expertise *[to make the decision] *because I'm not in the medical field... *[but] *my wife and I make the decision whether to allow them to go ahead' *[FM7, page 2]). Despite clearly stating this central role, many family members also admitted that they would usually take the advice of staff:

*'When the *[nurse] *said, "Would you have any objections if we send *[your mother] *to the hospital?", I said, "No. If you think that that's what should be done, I think that's what you should do.... I'm not a doctor. If you think that's the case, by all means just phone me and I'll be right there".' *[FM2, page 5]

## Discussion

Understanding resident and family member preferences about care is an essential ingredient to increasing satisfaction with care [16]. In this study on nursing home-acquired pneumonia, we captured the voices of both residents who were capable of making their own decisions in regards to their care and of family members speaking on behalf of residents who were incapable of expressing themselves. Both of these groups preferred that care be provided in the nursing home, although family members were more open to the idea of providing residents with hospital-based care. This preference is based on both their beliefs about pneumonia (not a health problem that must be cared for in hospital) and their assessment of 'good care'. For both groups of study participants, comfort and personalized care were the two most important components of care and were perceived as being more available in LTCFs.

Regarding treatment decision-making, family members believed that their preferences are regularly taken into account by LTCF staff, although many reported they would most likely take staff recommendations. Residents, on the other hand, felt that doctors should make treatment decisions, including locus of care. They were also much more hesitant than family members to express treatment preferences or criticize their care.

Other studies that have investigated resident preferences for care (such as [7] and [8]) have found a greater preference for hospital care. One plausible explanation for the discrepancy between the study findings may be that people tend to respond differently to questions about preferred locus of care if they are asked about hypothetical versus actual situations.

The role of the nursing home or LTCF is another factor that should be taken into account when developing programs to ensure patient- or resident-centred care. In response to the recent economic restructuring of health services in Canada, as well as changes in social values and demographic patterns, increasing numbers of older adults will receive care in a LTCF rather than in their own or a family member's home. In essence, these facilities will serve as both a person's home and a place where serious health care needs are met. The results of this study suggest that nursing home residents' values and beliefs about pneumonia do not elicit a strong desire for care in what Kleinman [9] refers to as the professional sector (hospital), but rather that they focus on the more personalized aspects of care that are traditionally associated with the popular sector (i.e. family and home). In LTCFs, where residents are personally known by staff and volunteers, the more personalized aspects of care, as well as biomedically appropriate treatments, are often available. This means that both disease- and illness-related changes in residents can be addressed when *in situ *care is provided. This was important for even those family members who indicated that an older person with pneumonia may receive better medical care in hospital.

There has been increased interest in measuring nursing home residents' satisfaction with care through surveys. However, little research has focused on understanding the reasons why residents prefer certain aspects of care or where such care is provided. Bowers et al. [18], in a qualitative study on nursing home residents' definition of quality care, identified three key components of care: care-as-service (instrumental aspects of care, such as how well, how quickly, and how consistently staff work is done), care-as-relating (affective aspects of care, such as staff-resident relationships and indications of affection), and care-as-comfort (whatever maintains or improves residents' physical comfort). In our study, residents' examples of good care included the two latter components, and family members discussed all three of them.

One possible reason why residents did not include instrumental aspects of care when discussing 'good care' could be their reluctance to criticize physicians. Family members, on the other hand, often assume the role of 'watchdog' for their loved ones, identifying and addressing problems with staff and facility administrators. Another reason may be that Bowers et al. focused on nursing home care in general, rather than on care for a specific health problem. It may be that people may evaluate normal, day-to-day care, such as the provision of meals and medications, differently than care received when they are ill. Our findings suggest that for acute care in the nursing home, residents may value comfort and caring related to their illness experience more strongly than the technical aspects of care that are more often associated with disease and hospital-based care.

A greater understanding of residents' and family members' preferences and satisfaction with treatment is crucial to developing viable models of resident-centred care. It may also play a vital role in enhancing resident cooperation with care plans, thereby improving health outcomes [19]. It is important, however, to make a clear distinction between individuals' preferences for locus of care and the level of involvement they want to have in treatment decision-making. Our findings, and those of O'Brien and colleagues [9], suggest that despite having specific treatment preferences, the majority of nursing home residents believe that doctors should make important treatment decisions. However, residents who do not want to play an active decision-making role may still want doctors to consider their preferences when faced with choices about their care [20].

Our findings are consistent with the literature in which older patients have been consistently shown to want less information and take a less active role in the treatment decision-making process [21-23]. In this study, we also found that residents were more hesitant to express their care preferences to physicians than family members. This may be related to their reluctance to criticize care (also noted in other studies; cf. [24] and [25]), or possibly the inability to evaluate their own care [25]. It may also be due to the role that family (informal) caregivers fill, which is to advocate on behalf of their loved ones in acute and long-term care facilities.

It is important to note that in the LTCF setting, research and patient-centred models acknowledge the pivotal role that family members play in decision-making and quality assessments. This research suggests that residents and family members may differ in their evaluations of care and their preferences for involvement in the decision-making process. Consequently, providing resident-focused care will require the understanding of both perspectives, particularly among people responding to actual illness episodes.

There are several limitations of this study. Although it is important to capture the voices of nursing home residents, interviewing residents can be challenging. Residents often have trouble expressing themselves and providing in-depth explanations, two key components of qualitative research. In some cases, residents conflate illness episodes over their lifetimes, making it hard to identify the specific context of their descriptions. Because of the limited number of participants and the cross-sectional nature of the study, we were not able to identify important variables that may influence resident or family member perspectives on care and determine if or how they change over the course of an illness. Our findings may be limited by the fact that no male nursing home residents were interviewed. However, it should be remembered that nursing home residents are mostly women. In the clinical trial from which residents were selected, 70% of the participants were female. This is similar to American profiles of nursing home residents where the ratio of women to men is approximately 3 to 1 [26]. Lastly, our study focused specifically on the views of residents and family members that were the primary decision makers in a resident's care decisions. The preferences of other family members and residents who cannot clearly express themselves may be different.

This study may be a good example of how qualitative studies can identify the underlying reasons for preferences around locus and type of care for older adults but not the prevalence of such views. To answer that question, quantitative surveys of larger numbers of individuals randomly selected from among LTCF residents and their families would be needed.

Building on this research, we would hope that future studies on this important topic explore the views of a broad range of residents and family members using a variety of methods, such as interviews, observation, and surveys, in order to more fully investigate factors that might influence the preferred locus of pneumonia care of residents and their family members. This includes individual factors (such as cognitive status, length of stay in the nursing home, past illness and hospital experiences) and contextual variables (such as quality of care and consistency of staff). It is also important to develop and use innovative research methodologies tailored for the nursing home setting to assess preferences for care and desired involvement in the decision-making process.

## Conclusion

The findings of this study have important implications for both future practice and research on pneumonia care for nursing home residents. Our work suggests that efforts to provide more on-site care are consistent with resident and family member preferences. The provision of acute care in nursing homes may become a more widely accepted option once additional work has been done to increase public awareness of the clinical skills and resources available in that setting, and of resident and family views supporting *in situ *care. Nursing homes may benefit from highlighting their ability to meet both disease- and illness-related facets of care, providing both state-of-the-art medical care as well as the personal attention and comfort measures that residents and family members consistently identified with good quality care. Although we are not recommending that nursing homes base their choice for locus of pneumonia care only on stated preferences by residents, we do suggest that even those seniors who do not want to be actively involved in making treatment decisions may have strong preferences for *in situ *care.

## Competing interests

The author(s) declare that they have no competing interests.

## Authors' contributions

All authors contributed to the design of the study and the writing of the manuscript. SCC collected the data, performed the data analysis, and drafted the manuscript. ML provided general supervision and assistance in the interpretation of the findings. LL supervised the data collection and analysis, and contributed to the interpretation of the findings. All authors read and approved the final manuscript.

## Pre-publication history

The pre-publication history for this paper can be accessed here:


